# Human-Identical Milk Oligosaccharides in Infant Formula: How Close Are We to Evidence-Based Benefit?

**DOI:** 10.1016/j.advnut.2026.100612

**Published:** 2026-03-03

**Authors:** Yarden Golan

**Affiliations:** Division of Nutritional Sciences, Cornell University, Ithaca, NY, United States

Human milk oligosaccharides (HMOs) are among the most intensively studied bioactive components of human milk, supported by strong mechanistic and observational evidence linking them to infant gut microbiota development, immune maturation, and host-microbe interactions [1]. Recent advances in biotechnology have enabled the industrial production of a limited number of these structures, referred to as human-identical milk oligosaccharides (HiMOs), and their incorporation into infant formula [2]. The previously published review by Shivakoti et al. [3] provides a timely and critical synthesis of randomized clinical trial evidence evaluating whether this innovation translates into meaningful health benefits for infants and young children.

A central conclusion of the review is that clinical evidence supporting the health benefits of HiMO supplementation remains limited [3]. Across trials conducted to date, HiMO-containing formulas consistently demonstrate safety, tolerability, and noninferior growth compared with standard formulas. However, evidence for reductions in morbidity, and particularly clinically relevant infectious outcomes, is inconsistent, modest in magnitude, and often derived from studies not powered for these endpoints [[2], [3], [4]]. The authors further highlight heterogeneity in the type of HMO used, infant age at intervention, and intervention duration, limiting the ability to draw overall conclusions [3]. Although several trials report shifts in gut microbiota composition or inflammatory biomarkers toward profiles observed in breastfed infants [5], such biological changes have not yet been reliably linked to improved clinical outcomes. Despite limited supporting evidence, multiple infant formula products now market the inclusion of HiMOs (marketed as HMOs) and are sold at a higher price than comparable nonsupplemented formulas. This review is therefore particularly important for clinicians and dietitians advising families on infant formula selection, as it helps determine whether the purported advantages of these formulas have a clinically evidence-based justification.

The review also highlights the need for more informative study designs and study populations. Most randomized trials to date have enrolled healthy, term infants who are exclusively formula-fed and living in high-income settings, where baseline risks for infection or growth impairment are relatively low. In such populations, detecting incremental benefits of formula modification is inherently challenging. Future well-designed randomized controlled trials powered for morbidity outcomes, particularly among infants at higher risk due to prematurity, undernutrition, or increased infectious exposure, will be essential to clarify whether HiMOs confer clinically meaningful advantages under these conditions [3].

Evidence from higher-risk populations, athough limited, further illustrates the complexity of HiMO effects. For example, in a trial conducted among infants with severe acute malnutrition, both the probiotic-containing arms outperformed the placebo in terms of weight gain; however, the greatest weight gain was observed in the probiotic-alone group rather than in the synbiotic arm receiving both *Bifidobacterium infantis* and HiMOs [6]. This finding suggests that, in this context, HiMO supplementation did not provide an additive growth benefit beyond that achieved by the probiotic itself. As HiMOs primarily function as prebiotics, their effects are likely contingent on the presence, abundance, and metabolic capacity of microbial taxa capable of utilizing them [7,8]. This observation underscores the importance of considering host-microbiome interactions when evaluating HiMO efficacy. The review reported that, in general, HiMO supplementation showed some beneficial effect on the infant microbiome, but also noted the variability in measuring this outcome between the studies, which should be improved in the future. Key, still open, questions include whether HiMOs exert greater effects when paired with specific probiotic strains, whether threshold concentrations are required to observe functional outcomes, and whether baseline microbial deficits modify responsiveness. Carefully designed synbiotic trials that integrate microbiome profiling and dose-response assessments of HiMO intake may be particularly informative, especially in populations with disrupted or immature gut microbiota.

In addition, human milk contains ∼200 structurally distinct HMOs [9], whereas current HiMO formulations typically include only one or a small number of structures, most commonly 2′-fucosyllactose. Several HMOs associated with protection against preterm morbidity or infectious outcomes are not yet available for large-scale production [10]. Whether health effects depend on individual HMOs, specific combinations, or the broader oligosaccharide mixture remains unknown, warranting caution in extrapolating from limited formulations to the complex biology of human milk.

From a policy perspective, the review findings reinforce the importance of maintaining appropriate expectations for HiMO supplementation [2,3]. Although formula innovations may benefit infants who cannot be fully breastfed, current evidence does not support HiMOs as a means to replicate the health benefits of breastfeeding. Continued investment in protecting, promoting, and supporting breastfeeding remains the most evidence-based strategy for improving infant health outcomes.

In summary, HiMOs represent a promising scientific advance, but the clinical evidence for HiMO supplementation to infant formula has not yet caught up with the biological rationale, which is based on observational studies with human milk. The review by Shivakoti et al. [3] provides a rigorous and appropriately cautious assessment of the current literature, emphasizing that despite consistent evidence of safety and tolerability, the biological activity of HiMOs and their impact on clinical outcomes remain incompletely defined, underscoring the need for further targeted studies.

## Author contributions

The sole author was responsible for all aspects of this manuscript.

## Declaration of generative AI and AI-assisted technologies in the manuscript preparation process

During the preparation of this work, the author used ChatGPT5.2 in order to edit some sentences. After using this tool/service, the author reviewed and edited the content as needed and takes full responsibility for the content of the published article.

## Funding

The author reported no funding received for this study.

## Conflict of interest

The author reports no conflicts of interest.

## References

[1] Duman H., Bechelany M., Karav S. (2024). Human milk oligosaccharides: decoding their structural variability, health benefits, and the evolution of infant nutrition. Nutrients..

[2] Hojsak I., Dinleyici E.C., van den Akker C.H.P., Domellöf M., Haiden N., Szajewska H. (2026). Technical review by the ESPGHAN Special Interest Group on Gut Microbiota and Modifications on the health outcomes of infant formula supplemented with manufactured human milk oligosaccharides. J. Pediatr. Gastroenterol. Nutr..

[3] Shivakoti R., Laughton B., Mandell J., Barrios-Tascon A., Rajendran R., Glashoff R. (2026). Limited evidence of benefits from clinical trials of human-identical milk oligosaccharides for infants. Adv. Nutr..

[4] Schönknecht Y.B., Moreno Tovar M.V., Jensen S.R., Parschat K. (2023). Clinical studies on the supplementation of manufactured human milk oligosaccharides: a systematic review. Nutrients.

[5] Parschat K., Melsaether C., Jäpelt K.R., Jennewein S. (2021). Clinical evaluation of 16-week supplementation with 5HMO-Mix in healthy-term human infants to determine tolerability, safety, and effect on growth. Nutrients.

[6] Nuzhat S., Hasan S.M.T., Palit P., Islam M.R., Mahfuz M., Islam M.M. (2023). Effects of probiotic and synbiotic supplementation on ponderal and linear growth in severely malnourished young infants in a randomized clinical trial. Sci. Rep..

[7] Ennis D., Shmorak S., Jantscher-Krenn E., Yassour M. (2024). Longitudinal quantification of *Bifidobacterium longum* subsp. *infantis* reveals late colonization in the infant gut independent of maternal milk HMO composition. Nat. Commun..

[8] Davis E.C., Castagna V.P., Sela D.A., Hillard M.A., Lindberg S., Mantis N.J. (2022). Gut microbiome and breast-feeding: Implications for early immune development. J. Allergy Clin. Immunol..

[9] Bode L. (2012). Human milk oligosaccharides: every baby needs a sugar mama. Glycobiology.

[10] Masi A.C., Embleton N.D., Lamb C.A., Young G., Granger C.L., Najera J. (2021). Human milk oligosaccharide DSLNT and gut microbiome in preterm infants predicts necrotising enterocolitis. Gut.

